# Cerebral attenuation on single-phase CT angiography source images: Automated ischemia detection and morphologic outcome prediction after thrombectomy in patients with ischemic stroke

**DOI:** 10.1371/journal.pone.0236956

**Published:** 2020-08-13

**Authors:** Paul Reidler, Daniel Puhr-Westerheide, Lukas Rotkopf, Matthias P. Fabritius, Katharina Feil, Lars Kellert, Steffen Tiedt, Jan Rémi, Thomas Liebig, Wolfgang G. Kunz

**Affiliations:** 1 Department of Radiology, University Hospital, LMU Munich, Munich, Germany; 2 Department of Neurology, University Hospital, LMU Munich, Munich, Germany; 3 German Center for Vertigo and Balance Disorders, LMU Munich, Munich, Germany; 4 Institute for Stroke and Dementia Research, LMU Munich, Munich, Germany; 5 Department of Neuroradiology, University Hospital, LMU Munich, Munich, Germany; University Hospital Basel, SWITZERLAND

## Abstract

**Objectives:**

Stroke triage using CT perfusion (CTP) or MRI gained importance after successful application in recent trials on late-window thrombectomy but is often unavailable and time-consuming. We tested the clinical value of software-based analysis of cerebral attenuation on Single-phase CT angiography source images (CTASI) as CTP surrogate in stroke patients.

**Methods:**

Software-based automated segmentation and Hounsfield unit (HU) measurements for all regions of the Alberta Stroke Program Early CT Score (ASPECTS) on CTASI were performed in patients with large vessel occlusion stroke who underwent thrombectomy. To normalize values, we calculated relative HU (rHU) as ratio of affected to unaffected hemisphere. Ischemic regions, regional ischemic core and final infarction were determined on simultaneously acquired CTP and follow-up imaging as ground truth. Receiver operating characteristics analysis was performed to calculate the area-under-the-curve (AUC). Resulting cut-off values were used for comparison with visual analysis and to calculate an 11-point automated CTASI ASPECTS.

**Results:**

Seventy-nine patients were included. rHU values enabled significant classification of ischemic involvement on CTP in all ten regions of the ASPECTS (each p<0.001, except M4-cortex p = 0.002). Classification of ischemic core and prediction of final infarction had best results in subcortical regions but produced lower AUC values with significant classification for all regions except M1, M3 and M5. Relative total hemispheric attenuation provided strong linear correlation with CTP total ischemic volume. Automated classification of regional ischemia on CTASI was significantly more accurate in most regions and provided better agreement with CTP cerebral blood flow ASPECTS than visual assessment.

**Conclusions:**

Automated attenuation measurements on CTASI provide excellent performance in detecting acute ischemia as identified on CTP with improved accuracy compared to visual analysis. However, value for the approximation of ischemic core and morphologic outcome in large vessel occlusion stroke after thrombectomy was regionally dependent and limited. This technique has the potential to facilitate stroke imaging as sensitive surrogate for CTP-based ischemia.

## Introduction

Single-phase CT angiography (CTA) is the most widely used technique to assess presence and location of large vessel occlusion (LVO) in the setting of acute ischemic stroke [1]. Considering the positive results of the 2015 thrombectomy trials, detection of LVO has become the most critical cornerstone in the diagnostic workflow to triage patients for endovascular thrombectomy (EVT) in order to achieve best clinical outcome [2].

After the positive results of the DAWN, DEFUSE 3, WAKE-UP and EXTEND trials, though, CT perfusion (CTP) and MRI have gained tremendous importance to identify late presenting patients who would benefit from EVT or intravenous thrombolysis (IVT) by determining properties of the ischemic core and penumbra [3–6]. However, advanced imaging methods are largely unavailable on a global scale, cost-intensive, time-consuming and need expertise, which is not available in a considerable amount of stroke treatment facilities [1, 7].

While noncontrast CT is the essential imaging method in stroke triage, traditional estimation of infarction extent using the Alberta Stroke Program Early CT Score (ASPECTS) suffers from high intra- and interreader variability, which makes it difficult to use for clinical decision making [8]. Yet, noncontrast CT is not sensitive to perfusion properties of ischemic tissue.

On the contrary, cerebral x-ray hypoattenuation on CTA source images (CTASI) reflects reduced blood supply rather than edema formation, based on the reduced contrast media uptake. Binary visual assessment of hypoattenuation in ASPECTS regions on CTASI has correlated well with final infarction and yielded higher sensitivity to detect ischemia than noncontrast CT [9–11]. Here, automated evaluation of CTASI would increase reliability in clinical application over visual ratings and further differentiate tissue status by quantitative cut-off values [12]. This technique might provide a surrogate for CTP and MRI parameters in case these methods are unavailable and thereby support therapy decisions in stroke patients.

Therefore, aim of our study was to examine the performance of automated attenuation measurements on CTASI to detect presence of regional ischemia and ischemic core as well as to predict final infarction in acute ischemic stroke.

## Materials and methods

### Study design and population

The study was approved by the institutional review board of the Ludwig-Maximilians-University Munich according to the Declaration of Helsinki of 2013 and requirement for written informed consent was waived. Patients with acute ischemic stroke due to anterior circulation large vessel occlusion were selected out of a consecutive cohort of 274 patients that were prospectively enrolled. Data analysis was performed retrospectively. All patients were treated with EVT at our institution between 2015 and 2017. In total, we selected seventy-nine patients.

We included patients with:

1. internal carotid artery, M1 or M2 segment artery occlusion,
2. complete noncontrast CT, CT angiography, and CTP imaging data.

We excluded patients with:

1. prior ischemia or intracranial mass, to ensure unbiased measurement of HU values,
2. pathology of the posterior circulation,
3. non-diagnostic imaging data.

All patients were previously reported in a study on automated attenuation measurements in ASPECTS regions on noncontrast CT [13].

### Image acquisition

Imaging protocol included noncontrast CT, arterial CTA and CTP. Examinations were performed using SOMATOM Definition AS+ and SOMATOM Definition Force scanners (Siemens Healthineers, Forchheim, Germany). CTP data were processed using the manufacturer’s software (syngo Neuro Perfusion CT, Siemens Healthineers, Forchheim, Germany) to generate perfusion maps.

CTA protocol included intravenous administration of 50mL iodinated contrast media, followed by a saline chaser of 40mL. Flow rate was 5mL/s. Imaging was performed in a single sweep from the aortic arch to the vertex with a bolus trigger of 100HU in the aortic trunk. Tube voltage was 120kV (SOMATOM Force) or 80kV (SOMATOM AS+) and tube current modulation (CareDose) was used. Collimation was 0.6mm. CTP was obtained with 100-mm scan coverage in the z-axis. 80kV voltage and 200 mAs current was applied. 35 mL of iodinated contrast agent (400 mg/mL) was administered intravenously at a flow rate of 5 mL/s, followed by a saline flush of 40 mL at 5 mL/s.

### Image analysis

Two blinded readers (radiology resident with 3 years [P.R.] and radiology attending with 6 years [W.G.K.] of experience in acute stroke imaging) determined overall ASPECTS on CTASI and ischemic involvement in ASPECTS regions on cerebral blood flow (CBF) maps in separate sessions for each modality. Regional ischemic core as deficit on cerebral blood volume (CBV) maps was determined by two blinded readers as described before [13]. In case of disagreement, consensus was reached in a separate session. Manual segmentation of total ischemic volume on CBF maps, ischemic core volume on CBV maps and final infarction on follow-up CT or MRI were performed using commercial software (OsiriX v.8.0.2, Pixmeo 2017). Final infarction was determined on follow-up imaging after 24-48h at CT or MRI for all ASPECTS regions and defined as present if ≥20% of the respective region was affected, according to other studies [14]. Collateral status was assessed on the scales by Tan et al. and Maas et al. in consensus by experienced readers ([W.G.K], [P.R.]) [10, 15].

### Automated analysis of tissue attenuation on CTA source images

**Fig 1:**
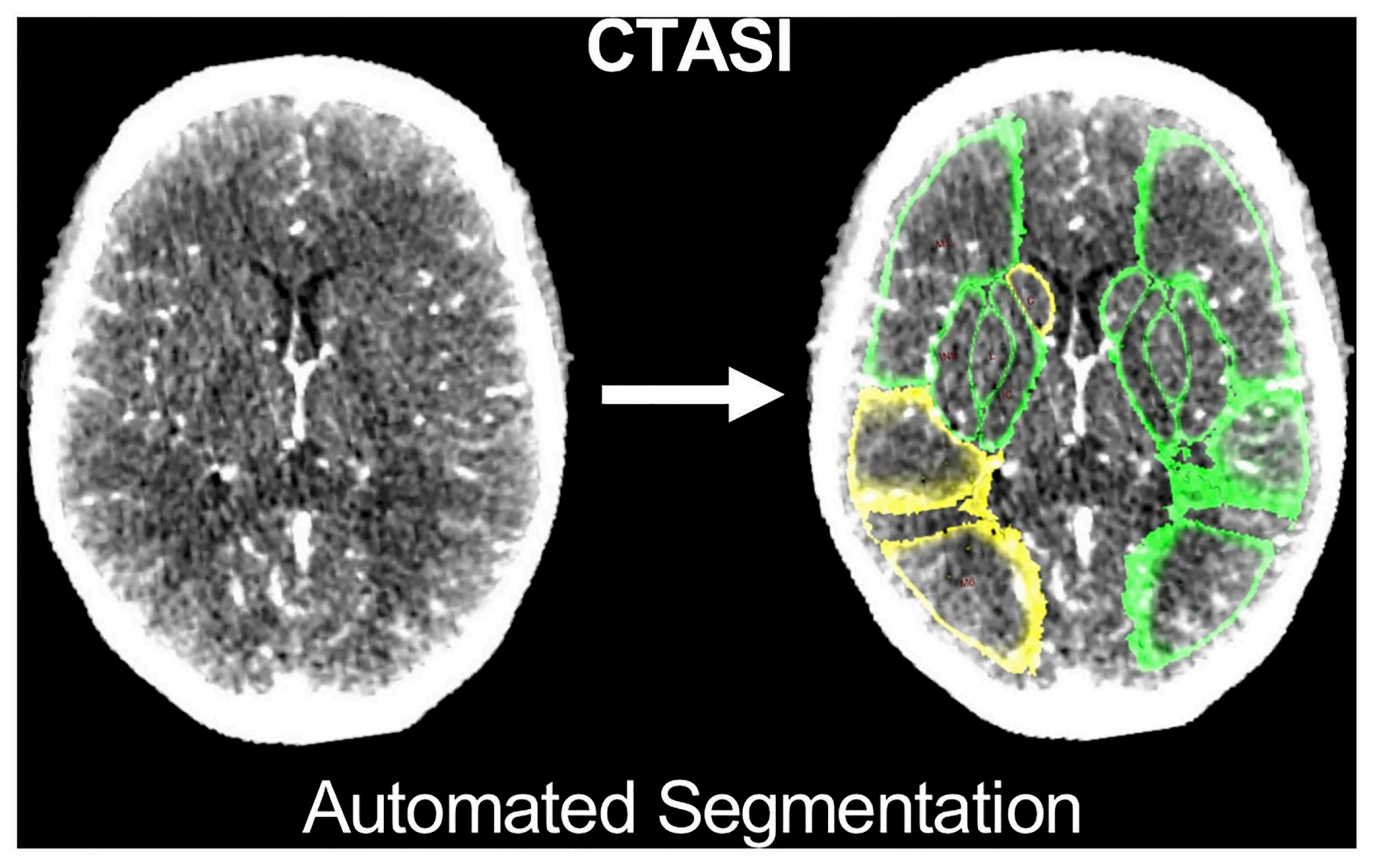
Segmentation of ASPECTS regions on CTASI. Abbreviations: ASPECTS, Alberta Stroke Program Early CT Score; CTASI, CT angiography source images; C, Caudate Nucleus; L, Lentiform Nucleus; C, Caudate; M1, M2 cortical regions of the ASPECTS. Colors indicate reduction in regional x-ray attenuation compared with the contralateral hemisphere (green = difference < 1 HU; yellow = difference ≥ 1 and < 3 HU; red = difference > 3 HU).

A population-based probabilistic ASPECTS atlas was created based on 221 normal noncontrast CT scans as previously reported [16] and implemented in a software prototype to calculate mean HU of each ASPECTS region (syngo.via Frontier, ASPECTS-Tool v1.2, Siemens Healthineers) [13, 17]. As the software was initially designed to analyze noncontrast CT data, correct segmentation was verified by expert readers ([P.R.] [W.G.K.]). An example of segmentation of ASPECTS regions on CTASI is provided in Fig 1. Further, relative HU (rHU) values were calculated for each ASPECTS region on CTASI as ratio of measured HU of the ischemic by the non-ischemic hemisphere (CTASI-rHU). Regional values for rHU were compared between regions with ischemic core, penumbra and without ischemic hypoperfusion. To calculate hemispheric CTASI-rHU as a comprehensive value, which integrates information of the whole MCA territory, the HU values of all ASPECTS regions were summed up separately for each hemisphere and divided ipsilateral by contralateral side.

### Statistical analysis

Analyses were performed in SPSS Statistics 23 (IBM, Armonk NY 2016, commercial software) and MedCalc version 18.10.2 (MedCalc Software, Ostend—Belgium, 2018, commercial software). All metric and ordinal variables are reported as median (interquartile range, IQR). Categorical variables are presented as number and percentage. Receiver operating characteristic (ROC) analyses using exact binomial confidence intervals compared the diagnostic performance of rHU value and Area-under-the-curve (AUC) values were calculated. Maximum Youden’s Index was used as indicator for best discriminative cut-off value to determine sensitivity and specificity of rHU values regarding regional ischemia, regional ischemic core and final infarction. Pearson’s correlation coefficient analysis tested the association of hemispheric CTASI-rHU values with total ischemic, ischemic core and final infarction volumes. Multivariate linear regression analysis was performed to determine association of hemispheric CTASI-rHU and collateral status. Measurements of inter-reader agreement including Cohen’s Kappa and intraclass correlation coefficient are provided in S1 and S2 Tables.

### Comparison of automated CTASI assessment

Youden-Index-derived cut-off values were used to determine regional tissue classification for overall ischemia and ischemic core, which was compared to consensus results of visual CTASI analysis with McNemar’s test. Same cut-off values were used to calculate an overall automated ASPECTS. From the cut-off values for regional ischemia we determined an ischemia weighted automated CTASI ASPECTS. From the cut-off values for ischemic core we determined an ischemic core weighted automated CTASI ASPECTS. Agreement of both parameters with visual CTASI ASPECTS, CBV and CBF ASPECTS as surrogate for core and overall ischemic extent were determined by intraclass correlation coefficient [18].

Further, AUC of measurements on CTASI for classification of the ischemic core were compared with prior results using noncontrast CT by the method of Hanley and McNeil [13, 19]. Difference of accuracy between measurements on CTASI and noncontrast CT were analyzed using McNemar’s Test.

## Results

### Patient characteristics

**Fig 2:**
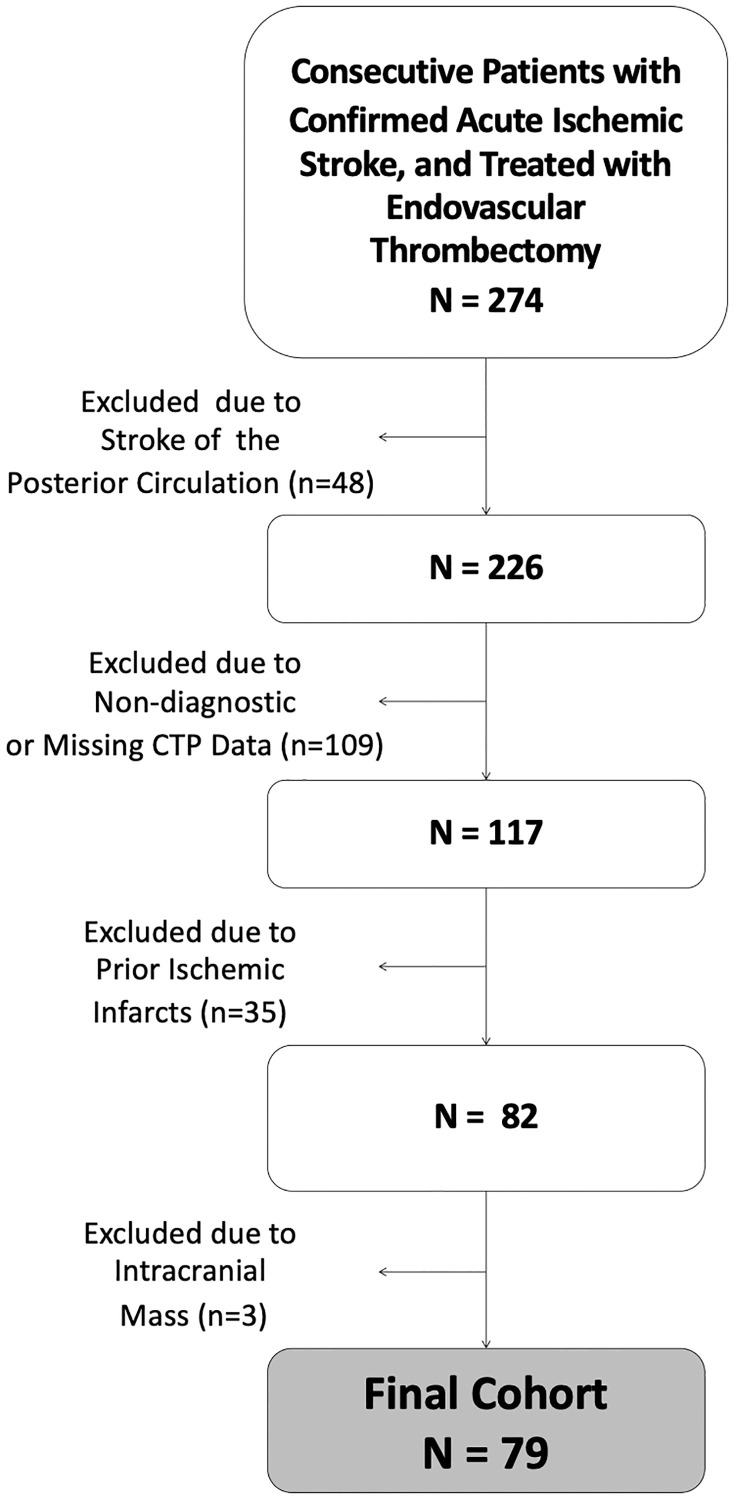
Flow-chart of patient selection. Abbreviations: CTP, CT perfusion.

**Fig 3:**
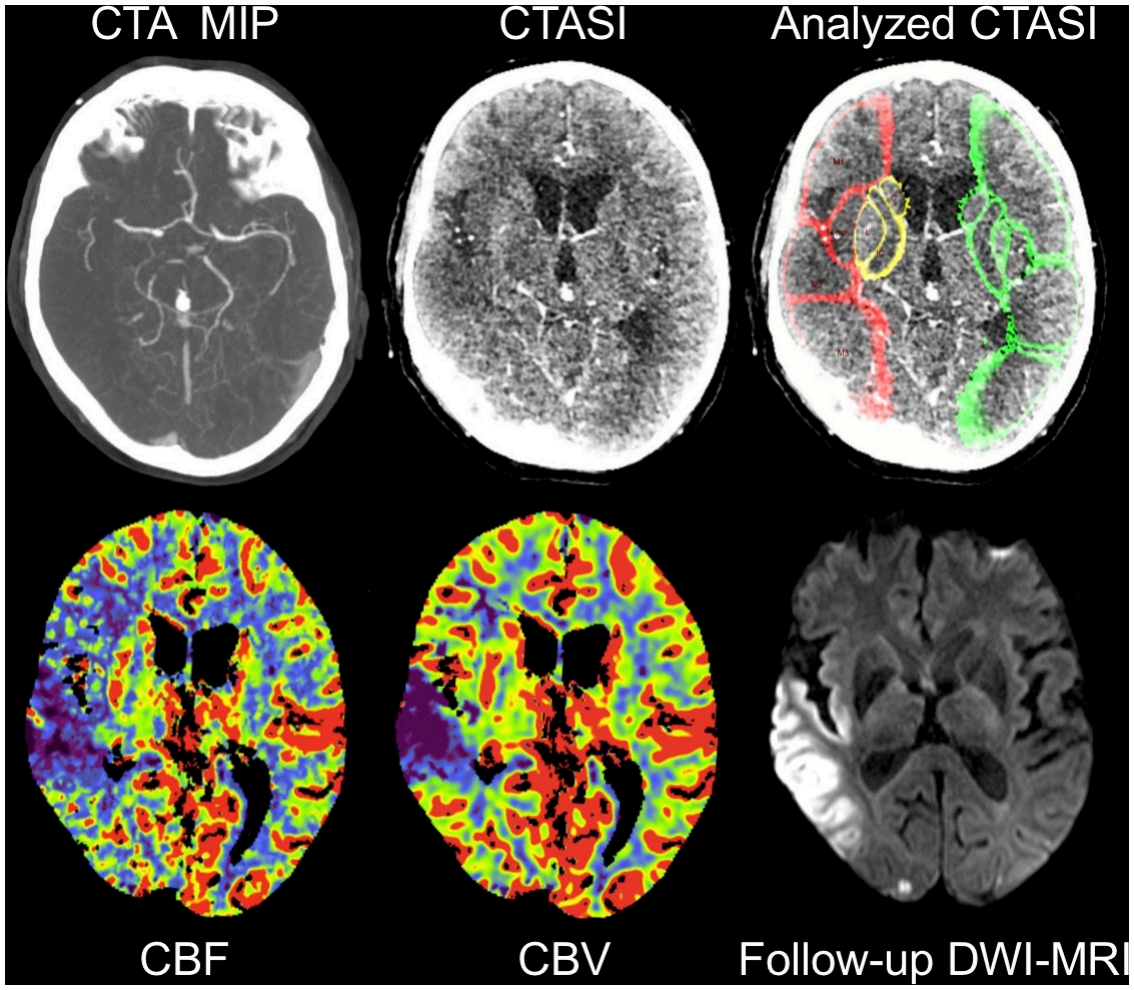
Case example of a 76-year-old male patient. CTA MIP shows a proximal M1 occlusion on the right side with consecutive cerebral blood flow and blood volume deficit on CTP. Noncontrast CT ASPECTS on admission was interpreted as 8 by both readers (not displayed). CTASI ASPECTS was 7 for reader 1 and 8 for reader 2. CTA source images without and after automated segmentation and attenuation measurements in ASPECTS regions presenting deficit of contrast uptake in the affected regions. Colors indicate reduction in regional x-ray attenuation compared with the contralateral hemisphere (green = difference < 1 HU; yellow = difference ≥ 1 and < 3 HU; red = difference > 3 HU). Notably, the analysis provides good match with ischemic hypoperfusion on CBF maps but overestimates infarction core on CBV and final infarction displayed on follow-up diffusion-weighted MRI after complete recanalization (mTICI 3). Abbreviations: CTA, CT angiography; MIP, maximum intensity projection; CTP, CT perfusion; CBF, cerebral blood flow; CBV, cerebral blood volume; DWI, diffusion-weighted imaging; ASPECTS, Alberta Stroke Program Early CT Score; mTICI, modified Treatment in Cerebral Ischemia Score.

Seventy-nine patients were included (37 female, 42 male, median age 76 [IQR: 62–82] and most frequently suffered from M1-occlusion (88.6%). See Fig 2 for detailed flow-chart. Median noncontrast CT ASPECTS was 8 [IQR: 8–10]. Median total ischemic volume was 143mL [IQR: 10-196mL] and median ischemic core volume was 17mL [IQR: 9-46mL]. Final infarction on follow-up imaging had a median of 19mL [IQR: 6-91mL]. Automated segmentation of ASPECTS regions on CTASI was successful for all cases as verified by expert readers’ consensus. Patient characteristics are displayed in Table 1. A case example is provided in Fig 3. Distribution of noncontrast CT ASPECTS and regional distribution of final infarction is presented in the S3 and S4 Tables.

**Table 1:** Patient characteristics of the study population.

Patient data (N = 79)		
Male sex	42	(53.2%)
Female sex	37	(47.8%)
Median Age	76	(64-82)
Time from symptom onset to CT (min)	82	(65-125)
NIHSS on admission	14	(9-17)
90-day mRS*	4	(2-6)
**Imaging data**		
Noncontrast CT-ASPECTS	8	(8-10)
CTASI ASPECTS	6	(3-8)
CBF-ASPECTS	3	(2-4)
CBV-ASPECTS	7	(6-8)
Total ischemic volume [mL]	143	(108-196)
Ischemic core volume [mL]	17	(9-46)
Mismatch volume [mL]	112	(70-151)
Final infarction volume [mL]	19	(6-91)
Occlusion location		
ICA	21	(26.6%)
Carotid T	18	(22.8%)
M1 segment of MCA	70	(88.6%)
M2 segment of MCA	20	(25.3%)
Follow-up imaging method		
CT	52	(65.8%)
MRI	27	(34.2%)
Reperfusion after Thrombectomy		
mTICI 0	11	(13.9%)
mTICI 1	1	(1.3%)
mTICI 2a	5	(6.3%)
mTICI 2b	38	(48.1%)
mTICI 3	24	(30.4%)

Values presented are count (percentage) for categorical and median (interquartile range) for ordinal or continuous variables. All volumes are presented in mL. ASPECTS indicates Alberta Stroke Program Early CT Score; CBF / CBV, cerebral blood flow / volume; CTASI, CT angiography, source images; ICA, internal carotid artery; MCA, middle cerebral artery; and NIHSS, National Institutes of Health Stroke Scale; mRS, modified Rankin Scale; mTICI, modified Treatment in Cerebral Ischemia Score. * 90-day mRS available for 58 patients.

#### Analysis of rHU values

rHU values were significantly different between regions of penumbra or ischemic core compared to regions without ischemic deficit, except for M5 cortex. Insula, M1 M2 and M6 provided difference between core and penumbra with p<0.05, however, after correction for multiple comparisons with Bonferroni’s method no significant difference could be observed. Detailed results are displayed in Table 2.

**Table 2:** CTASI rHU values in ASPECTS regions with CT Perfusion–based ischemic core or penumbra or without CT perfusion–based hypoperfusion.

Location	rHU for ASPECTS Regions with CT Perfusion–based Core		n	rHU for ASPECTS Regions with CT Perfusion–based Penumbra		n	rHU for ASPECTS Regions without CT Perfusion–based Hypoperfusion		n	P Value Penumbra vs. Core	P Value No Deficit vs. Core	P Value No Deficit vs. Penumbra
C	0.828	(0.808–0.861)	19	0.875	(0.821–0.924)	20	0.963	(0.936–0.985)	40	0.067	<**0.001**	<**0.001**
IC	0.890	(0.876–0.904)	22	0.882	(0.860–0.919)	16	0.968	(0.936–0.997)	41	0.68	<**0.001**	<**0.001**
INS	0.785	(0.734–0.836)	46	0.831	(0.779–0.876)	29	0.970	(0.936–0.986)	4	0.04	<**0.001**	0.006
L	0.799	(0.774–0.834)	27	0.817	(0.796–0.900)	13	0.951	(0.910–0.986)	39	0.10	<**0.001**	<**0.001**
M1	0.889	(0.847–0.930)	21	0.920	(0.896–0.952)	49	0.966	(0.941–0.993)	9	0.01	<**0.001**	**0.003**
M2	0.852	(0.828–0.885)	30	0.901	(0.866–0.930)	46	0.971	(0.946–1.028)	3	0.006	0.006	0.009
M3	0.946	(0.929–0.985)	11	0.966	(0.942–0.989)	52	1.000	(0.988–1.017)	16	0.23	**0.001**	<**0.001**
M4	0.945	(0.921–0.969)	19	0.965	(0.946–0.983)	45	0.978	(0.959–1.013)	15	0.07	**0.003**	0.04
M5	0.912	(0.882–0.947)	32	0.915	(0.893–0.938)	44	1.009	(0.928–1.028)	3	0.67	0.082	0.08
M6	0.937	(0.920–0.945)	17	0.975	(0.939–0.987)	50	0.995	(0.983–1.040)	12	0.01	<**0.001**	**0.001**

rHU values are displayed as median (interquartile range). Ischemic core was defined as ischemic change on the parametric cerebral blood flow map as well as cerebral blood volume map. Penumbra was defined as ischemia on the cerebral blood flow maps without matching changes on cerebral blood volume maps. C indicates caudate nucleus; IC, internal capsule; INS, insula; L, lentiform nucleus; M1-M6, cortical regions of the ASPECTS score; rHU, relative Hounsfield Units; ASPECTS, Alberta Stroke Program Early CT Score. P Values <0.05 indicate statistical significance. Bold values indicate significance after correction for multiple comparisons using Bonferroni’s method.

#### Classification of CT perfusion-based regional ischemia

**Fig 4:**
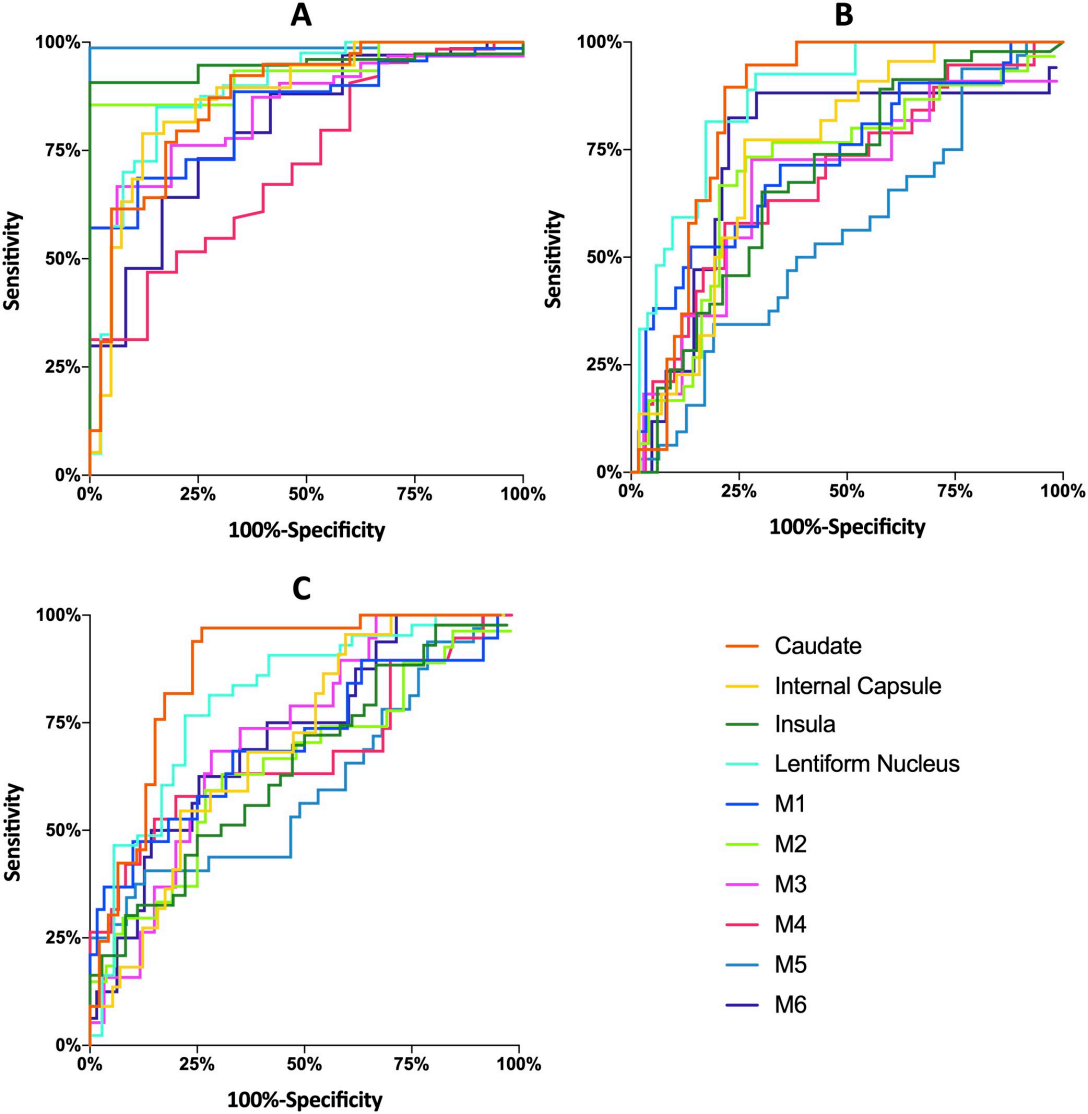
Receiver operating characteristic curves for classification of (A) CT perfusion-based ischemia, (B) CT perfusion-based ischemic core and (C) regional final infarction by CTASI-rHU for all Alberta Stroke Program Early CT Score regions. Abbreviations: M1-M6, M1-6 Cortices of the Alberta Stroke Program Early CT Score; CTP, CT perfusion; CTASI, CT angiography source images; rHU, relative Hounsfield Units.

CTASI-rHU values were able to perform significant classification with excellent results in all ten ASPECTS regions. AUC values varied from 0.72 to 0.99 with best performance in M5-cortex (AUC 0.99, p<0.001, sensitivity: 99%, specificity: 100%) and subcortical regions (each p<0.001, sensitivity: 79 to 92%, specificity: 68 to 100%). Results are displayed in Table 3, ROC curves are provided in Fig 4.

**Table 3:** ROC analysis of CTASI-rHU values for the classification of indicated parameters.

Location	AUC (95% CI)		P Value	Youden’s Index	Associated Cut-Off Value (rHU)	Associated Sensitivity	Associated Specificity
**Classification of Regional CT Perfusion-Based Ischemia**							
C	0.87	(0.78–0.94)	<0.001	0.60	0.947	92% (36/39)	68% (27/40)
IC	0.87	(0.78–0.94)	<0.001	0.67	0.919	79% (30/38)	88% (35/41)
INS	0.95	(0.87–0.98)	<0.001	0.91	0.972	91% (68/75)	100% (4/4)
L	0.89	(0.80–0.95)	<0.001	0.70	0.873	85% (34/40)	85% (33/39)
M1	0.84	(0.74–0.91)	<0.001	0.58	0.933	69% (48/70)	89% (8/9)
M2	0.93	(0.86-0-98)	<0.001	0.86	0.941	86% (65/76)	100% (3/3)
M3	0.85	(0.75–0.92)	<0.001	0.60	0.976	67% (42/63)	94% (15/16)
M4	0.72	(0.61–0.81)	0.002	0.34	0.953	47% (28/64)	87% (13/15)
M5	0.99	(0.94–1.00)	<0.001	0.99	0.991	99% (75/76)	100% (3/3)
M6	0.80	(0.69–0.88)	<0.001	0.48	0.977	64% (43/67)	83% (10/12)
**Classification of Regional CT Perfusion-Based Ischemic Core**							
C	0.85	(0.75–0.92)	<0.001	0.68	0.884	95% (18/19)	73% (44/60)
IC	0.75	(0.64–0.84)	<0.001	0.51	0.904	77% (17/22)	74% (42/57)
INS	0.69	(0.57–0.79)	0.003	0.35	0.803	65% (30/46)	70% (23/33)
L	0.87	(0.77–0.93)	<0.001	0.64	0.837	82% (22/27)	83% (43/52)
M1	0.72	(0.61–0.82)	0.001	0.39	0.889	52% (11/21)	86% (51/58)
M2	0.70	(0.59-0-80)	0.001	0.47	0.878	73% (22/30)	74% (36/49)
M3	0.67	(0.56–0.78)	0.08	0.45	0.953	73% (8/11)	72% (49/68)
M4	0.68	(0.57–0.78)	0.01	0.36	0.945	58% (11/19)	78% (47/60)
M5	0.55	(0.43–0.66)	0.45	0.17	0.958	94% (31/32)	23% (11/47)
M6	0.75	(0.63–0.84)	0.001	0.60	0.966	82% (14/17)	77% (48/62)
**Prediction of Final Infarction**							
C	0.87	(0.78–0.94)	<0.001	0.71	0.923	97% (32/33)	73% (34/46)
IC	0.71	(0.59–0.80)	<0.001	0.36	0.955	96% (21/22)	40% (23/57)
INS	0.65	(0.54–0.76)	0.01	0.24	0.781	49% (21/43)	75% (27/36)
L	0.81	(0.71–0.89)	<0.001	0.55	0.876	77% (33/43)	78% (28/36)
M1	0.71	(0.60–0.81)	0.07	0.37	0.880	47% (9/19)	90% (54/60)
M2	0.65	(0.54–0.76)	0.02	0.32	0.859	59% (16/27)	73% (38/52)
M3	0.72	(0.61–0.82)	<0.001	0.40	0.957	68% (13/19)	72% (43/60)
M4	0.68	(0.57–0.78)	0.03	0.38	0.945	58% (11/19)	80% (48/60)
M5	0.60	(0.49–0.71)	0.13	0.28	0.887	41% (13/32)	87% (41/47)
M6	0.72	(0.61–0.81)	0.002	0.37	0.942	63% (10/16)	75% (48/63)

rHU was defined as the ratio of regional x-ray attenuation measurements of the ischemic to the non-ischemic hemisphere. Ischemic core was defined as ischemic change on the parametric cerebral blood flow map as well as cerebral blood volume map. Sensitivity and specificity for the indicated cut-off value are presented as percentage and numbers as raw data in parentheses. AUC indicates area under the curve values; C, caudate nucleus; CI, confidence interval; IC, internal capsule; INS, insula; L, lentiform nucleus; M1-M6, cortical regions of the ASPECTS score; rHU, relative Hounsfield Units; and ROC, receiver operating characteristics. P Values <0.05 indicate statistical significance.

### Classification of CT perfusion-based ischemic core

CTASI-rHU values were able to perform significant classification of ischemic core in all ASPECTS regions except M3- and M5-cortex with best results in subcortical areas Caudate, internal capsule and lentiform nucleus (AUC: 0.75 to 0.87, each p<0.001, sensitivity: 77% to 95%, specificity: 73% to 83%). Results are displayed in Table 3; ROC curves are provided in Fig 4.

Comparison of AUC and accuracy between measurements on CTASI and noncontrast CT are provided in S5 Table. While no significant difference in AUC of both methods can be observed, resulting cut-off values presented significantly better accuracy of CTASI measurement in the regions internal capsule, lentiform nucleus, M2, M3 and M5 cortices, similar accuracy in the region insula, M1, M5 and M6 and better accuracy of noncontrast CT in the region caudate.

#### Classification of final infarction

CTASI-rHU values were able to significantly predict final infarction in all ASPECTS regions except M1- and M5-cortex with best results in subcortical areas Caudate, internal capsule and lentiform nucleus (AUC: 0.71 to 0.87, each p<0.001, sensitivity: 77% to 97%, specificity: 73% to 78%). Results are displayed in Table 3, ROC curves are provided in Fig 4. A subgroup analysis of patients with successful (mTICI 2b-3) or unsuccessful (mTICI 0-2a) reperfusion after EVT is provided in S6 Table.

#### Correlation of hemispheric CTASI-rHU with lesion extent

**Fig 5:**
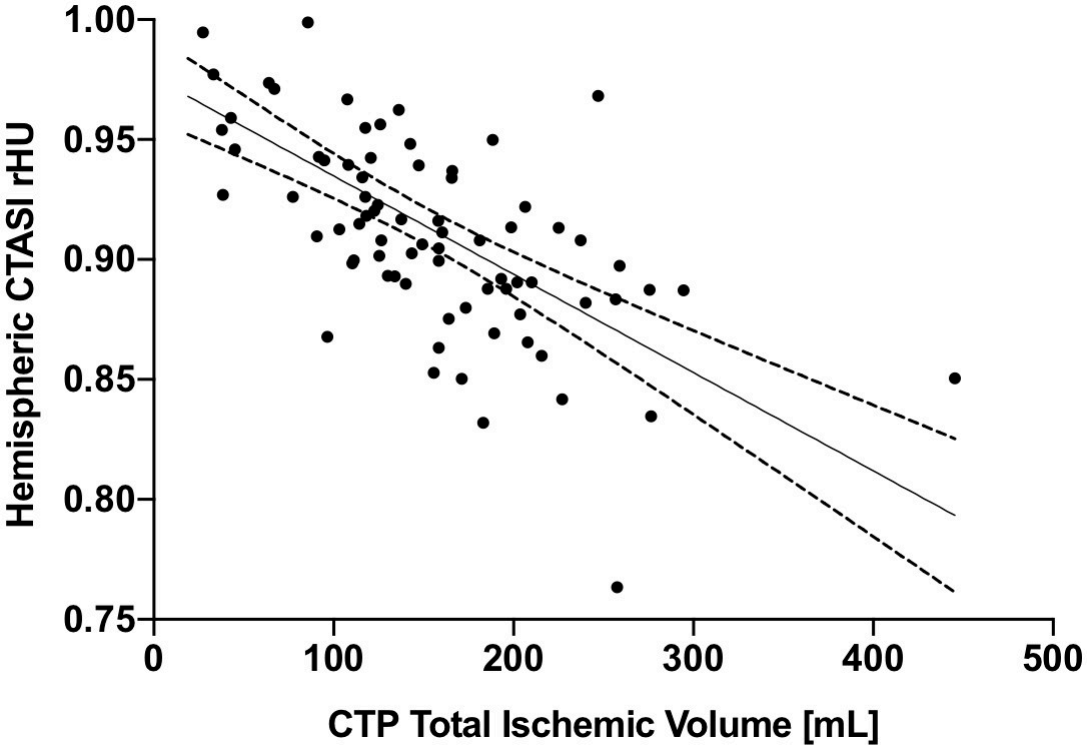
Linear correlation of hemispheric CTASI-rHU and CTP-based total ischemic volume. Graph presents scatter plot, trend line and 95% confidence interval. Abbreviations: CTASI, CT angiography source images; rHU, relative Hounsfield Units; CTP, CT perfusion.

In linear correlation analysis, hemispheric CTASI-rHU values presented a strong association with total ischemic volume (Pearson correlation coefficient: -0.661, p<0.001) while presenting medium, yet significant strengths of association for ischemic core volume (Pearson correlation coefficient: -0.317, p = 0.004) and final infarction volume (Pearson correlation coefficient: -0.289, p<0.023). Results are displayed in Table 4, scatter plot and trendline for association with total ischemic volume are provided in Fig 5. Distribution of collateral scores are displayed in S7 Table. In multivariate linear regression analysis, no independent associations of collateral status with hemispheric CTASI-rHU were shown as displayed in the S8 and S9 Tables.

**Table 4:** Correlation of hemispheric CTASI rHU with CTP parameters and final infarction volume.

Hemispheric CTASI rHU (N = 79)	Total Ischemic Volume [mL]	Ischemic Core Volume [mL]	Final Infarction Volume [mL]
**Pearson Correlation Coefficient (95%-CI)**	-0.661 (-0.770 –-0.515)	-0.317 (-0.503 –-0.103)	-0.289 (-0.503 –-0.042)
**P-Value**	<0.001	0.004	0.023

Pearson’s correlation coefficient was calculated for the indicated parameters. Abbreviations: CTASI, CT angiography source images; rHU, relative Hounsfield units; CTP, CT perfusion, CI, Confidence interval. P values <0.05 indicate statistical significance.

#### Comparison of automated and visual analysis of CTASI

Automated classification of regional ischemia was significantly more accurate than visual assessment for all regions except for par results in the region caudate and lentiform nucleus. Classification of ischemic core presented mixed results with improved accuracy of automated assessment in M1 and M2 cortices, equal results in caudate, internal capsule, insula, lentiform nucleus and M4 and M6 cortices, and better results for visual assessment in M3 and M5 cortices. Detailed results are displayed in Table 5.

**Table 5:** Comparison of visual and automated analysis of CTASI and for the classification of regional ischemia and ischemic core.

Location	Visual Classification Sensitivity / Specificity		Visual Accuracy	Automated Classification Sensitivity / Specificity		Automated Accuracy	P Value
**Classification of Regional CT Perfusion-based Ischemia**							
C	72% (28/39)	83% (33/40)	77%	92% (36/39)	68% (27/40)	80%	0.50
IC	61% (23/38)	83% (33/41)	71%	79% (30/38)	88% (35/41)	86%	<0.001
INS	95% (71/75)	75% (3/4)	94%	91% (68/75)	100% (4/4)	91%	0.50
L	73% (29/40)	82% (32/39)	77%	85% (34/40)	85% (33/39)	85%	0.03
M1	47% (33/70)	89% (8/9)	52%	69% (48/70)	89% (8/9)	71%	<0.001
M2	67% (51/76)	100% (3/3)	68%	86% (65/76)	100% (3/3)	86%	<0.001
M3	19% (12/63)	94% (15/16)	34%	67% (42/63)	94% (15/16)	72%	<0.001
M4	28% (18/64)	93% (14/15)	41%	47% (28/64)	87% (13/15)	52%	0.004
M5	49% (37/76)	100% (3/3)	51%	99% (75/76)	100% (3/3)	99%	<0.001
M6	24% (16/67)	100% (12/12)	46%	64% (43/67)	83% (10/12)	67%	<0.001
**Classification of Regional CT Perfusion-based Ischemic Core**							
C	100% (19/19)	73% (44/60)	80%	95% (18/19)	73% (44/60)	78%	1.00
IC	77% (17/22)	77% (44/57)	77%	77% (17/22)	74% (42/57)	75%	0.50
INS	98% (45/46)	12% (4/33)	62%	65% (30/46)	70% (23/33)	67%	0.13
L	82% (22/27)	73% (38/52)	76%	82% (22/27)	83% (43/52)	82%	0.06
M1	62% (13/21)	64% (37/58)	63%	52% (11/21)	86% (51/58)	78%	<0.001
M2	73% (22/30)	41% (20/49)	53%	73% (22/30)	74% (36/49)	73%	<0.001
M3	46% (5/11)	88% (60/68)	82%	73% (8/11)	72% (49/68)	72%	0.008
M4	37% (7/19)	80% (48/60)	70%	58% (11/19)	78% (47/60)	73%	0.25
M5	72% (23/32)	70% (33/47)	71%	94% (31/32)	23% (11/47)	53%	<0.001
M6	41% (7/17)	86% (53/62)	76%	82% (14/17)	77% (48/62)	78%	0.25

Ischemic core was defined as ischemic change on the parametric cerebral blood flow map as well as cerebral blood volume map. Presence of hypoattenuation on CTASI as well as CT perfusion-based core and overall ischemia topography was determined by expert reader consensus. Comparison of test Accuracy was performed with McNemar’s Test. C indicates caudate nucleus; CI, confidence interval; IC, internal capsule; INS, insula; L, lentiform nucleus; M1-M6, cortical regions of the Alberta Stroke Program Early CT Score; CTASI, CT angiography source images. P Values <0.05 indicate statistical significance.

The ischemia weighted and core weighted automated CTASI ASPECTS presented good agreement with visual CTASI ASPECTS (ICC [95%-CI]: 0.79 [0.67–0.89] and 0.79 [0.66–0.86]). Only the ischemia weighted CTASI ASPECTS presented good agreement with CBF ASPECTS (ICC [95%-CI]: 0.77 [0.64–0.85]). Agreement with CBV ASPECTS was moderate for all parameters. Notably, visual CTASI only reached moderate agreement with CBF and CBV ASPECTS (ICC [95%-CI]: 0.58 [0.35–0.73] and 0.57 [0.33–0.73]). Detailed results are displayed in S10 Table.

## Discussion

Our study represents the first quantitative analysis linking automated measurements of HU-values on CTASI to CTP-based tissue status. rHU in ASPECTS regions on CTASI provided excellent detection of CTP-based ischemic tissue, however presented only limited value in the detection of ischemic core and final infarction. A strong linear relationship was shown between combined hemispheric CTASI-rHU and total ischemic volume.

The recent DAWN, DEFUSE 3, WAKE-UP and EXTEND trials have been pushing the time-boundaries of EVT and IVT, providing ample evidence for therapy benefit in late presenting stroke patients [3–6]. Still, patients in late or unknown time-windows require careful imaging-based triage as positive evidence, so far, is only available for patients with small ischemic core and positive mismatch profile. In stroke treating facilities without CTP or MRI imaging capacity or experience, this might cause diagnostic uncertainty in the decision to transfer patients to a comprehensive center where repeated imaging on arrival can lead to substantial time delays as reported by Froehler et al. [20]. A recent randomized trial reported time-savings of around 30min for noncontrast CT / CTA compared to MRI triage for admission to groin puncture and reperfusion [21]. Even the additional use of CTP would produce a substantial delay of around 15min, which is associated with worse clinical outcome and reduced cost-effectiveness in patients treated with EVT [7, 22]. Apart from time-savings, the reliance on a noncontrast CT / CTA protocol would also reduce patient disbenefit by lowering radiation dose and administered contrast media especially in modern scanners with 128-slices or more [23, 24].

Our used, modern CTA protocols seem to overestimate the ischemic core [25, 26], as especially coverage in the later arteriovenous phase effects best correlation with final infarction [27]. Accordingly, attenuation measurements on CTASI in our study present a better correlation with total ischemic volume, than ischemic core volume. Here, we provide novel evidence that quantitative analysis of hypoattenuation on modern CTA protocols is directly linked to ischemic topography on CTP imaging as defined by the ASPECTS framework. Overall, this presents a strong rationale for the technique’s use as fast stroke screening due to the marked performance in overall ischemia detection. Further, rHU values can provide a surrogate of ischemic extent in LVO stroke patients in case CTP or MRI are unavailable. However, our study has shown limitations for core estimation in single-phase CTA protocols. This needs to be taken into account as estimation of the infarction core is an important determinant for therapy decision, and overestimation, which might keep patients from receiving treatment, needs to be avoided. As we assume similar performance of hypoattenuation-detection in later-timed CTA protocols, there is high potential of this technique in the combined use with later acquisitions of multi-phase CTA data. This could provide further detail on the ischemic core and mismatch profile, which needs to be examined in further studies [28]. Notably, an advanced machine learning approach by Sheth et al. reached substantial correlation of single-phase CTASI analysis with CTP derived numerical ischemic core volumes [29]. This indicates future potential of advanced CTASI analysis to aid in clinical decision making, even using single-phase protocols. Already, rHU measurements on CTASI were able to provide additional value for classification of ischemic core regions with higher AUC and significantly improved accuracy for most regions as same measurements on noncontrast CT [13].

Clearly, we have found regional differences in classification performance. While the approach produced high sensitivity /specificity for ischemia detection in almost all regions, classification of ischemic core and prediction of final infarction presented best performance in subcortical regions. The latter was driven by patients with successful reperfusion. Variability of recanalization in patients with incomplete reperfusion (mTICI:0-2a) might explain inferior results in this subgroup, which suffers from its small sample size (n = 17).

Compared to visual analysis, automated CTASI assessment has proven to be more sensitive for ischemic changes and provided better agreement with CBF ASPECTS as surrogate for total ischemic extent. Agreement with CTP was even higher than the moderate agreement between readers. Although some studies report higher interreader agreement for visual CTASI, there is high variability of visual assessment between studies, underlining the benefit of a standardized automated approach [8, 12, 30].

Already, CTA is a cost-effective imaging method for stroke triage [31] and its information gain of single-phase CTA is not yet exploited by our automated approach. Visual assessment of leptomeningeal collaterals assessed on CTA have proven among the strongest predictors of clinical outcome, even using only single-phase imaging [32–34]. Also, our approach did not rely on deep learning techniques as used before with voxel-wise AUC of up to 0.93 for infarction classification [35], indicating potential of improvement by further incorporating advanced image processing or even clot detection [36]. Advantageously, our analysis of HU values presents a reproducible and coherent imaging biomarker and the scaffold of regional analysis by ASPECTS regions allows integration with other imaging modalities.

Limitations of this study include: First, we provide only a limited patient set with rather small ischemic cores. Therefore, validation using larger cohorts and a greater set of lesion extent is warranted. Second, the results from our institutions CT protocol are not generalizable, as significant variability in hypoattenuation across protocols was shown before [25]. Third, we only relied on one vendor platform (Siemens Healthineers), yet due to the normalization of rHU values over the healthy hemisphere we assume similar results on other platforms. Fourth, we relied on visual classification of regional CTP status as, to the best of our knowledge, no software solution for automated classification in ASPECTS regions is currently available for this modality.

In conclusion, automated analysis of CTA source images is a promising tool for simplified and accelerated imaging in large vessel occlusion stroke, which provides excellent detection of ischemia and can further aid in estimation of the ischemic core and final infarction.

## Supporting information

S1 Table Inter-reader agreement for the regional presence of CTP defined ischemia. (DOCX)

S2 Table Inter-reader agreement for CTASI ASPECTS. (DOCX)

S3 Table Distribution of noncontrast CT ASPECTS. (DOCX)

S4 Table Regional distribution of final infarction in ASPECTS regions. (DOCX)

S5 Table Comparison of automated rHU measurements on CTASI and noncontrast CT for the classification of regional ischemic core. (DOCX)

S6 Table ROC analysis of CTASI-rHU values for the classification of indicated parameters. (DOCX)

S7 Table Distribution of collateral scores. (DOCX)

S8 Table Linear regression analysis for the association of hemispheric CTASI-rHU and collateral status by Maas et al. (DOCX)

S9 Table Linear regression analysis for the association of hemispheric CTASI-rHU and collateral status by Tan et al. (4). (DOCX)

S10 Table Agreement of automated and visual CTASI ASEPCTS. (DOCX)

S1 File Supporting material references. (DOCX)
