# Supplementary material for: Cerebral attenuation on single-phase CT angiography source images: Automated ischemia detection and morphologic outcome prediction after thrombectomy in patients with ischemic stroke
Source: PLoS One. 2020 Aug 13;15(8):e0236956. doi: 10.1371/journal.pone.0236956 (PMC7425881; doi:10.1371/journal.pone.0236956)
Supplement: S1 Table — (DOCX) [file pone.0236956.s001.docx]

| **S1 Table. Inter-Reader Agreement for the Regional Presence of CTP Defined Ischemia** | | | |
| --- | --- | --- | --- |
| **N=79** | **Kappa** | **(95% CI)** | **Interpretation** |
| C | 0.70 | (0.54-0.85) | substantial |
| IC | 0.72 | (0.57-0.87) | substantial |
| INS | 0.74 | (0.39-1.00) | substantial |
| L | 0.75 | (0.60-0.89) | substantial |
| M1 | 0.50 | (0.23-0.78) | moderate |
| M2 | 0.74 | (0.39-1.00) | substantial |
| M3 | 0.67 | (0.46-0.88) | moderate |
| M4 | 0.63 | (0.40-0.86) | moderate |
| M5 | 0.39 | (0.15-0.92) | poor |
| M6 | 0.51 | (0.23-0.79) | moderate |
| Measurements of inter-reader agreement for the presence of ischemia on CT perfusion imaging using Cohen´s Kappa. Kappa Interpretation: <0.2 – slight, 0.21-0.4 – fair, 0.41-0.6 – moderate, 0.61-0.8 – substantial, 0.81-1.0 – almost perfect (1). CTP indicates CT Perfusion; CI, confidence interval, C, caudate nucleus; CI, confidence interval; IC, internal capsule; INS, insula; L, lentiform nucleus; M1-M6, cortical regions of the Alberta Stroke Program early CT score; CTASI, CT angiography source images. | | | |
