# Supplementary material for: Cerebral attenuation on single-phase CT angiography source images: Automated ischemia detection and morphologic outcome prediction after thrombectomy in patients with ischemic stroke
Source: PLoS One. 2020 Aug 13;15(8):e0236956. doi: 10.1371/journal.pone.0236956 (PMC7425881; doi:10.1371/journal.pone.0236956)
Supplement: S2 Table — (DOCX) [file pone.0236956.s002.docx]

| **S2 Table. Inter-Reader Agreement for Visual ASPECTS on CTASI** | | | |
| --- | --- | --- | --- |
|  | **Intraclass Correlation Coefficient** | **(95%-CI)** | **Interpretation** |
| **N=79** | | |  |
| Average measures | 0.60 | (0.27-0.77) | moderate |
| Measurements of inter-reader agreement for CTASI ASPECTS among both readers using intraclass correlation coefficient. ICC Interpretation: <0.5 – poor, 0.5-0.75 – moderate, >0.75 – good (2). ASPECTS indicates Alberta Stroke Program Early CT Score; CTASI; CT angiography source images; CI, confidence interval. | | | |
