# Supplementary material for: Cerebral attenuation on single-phase CT angiography source images: Automated ischemia detection and morphologic outcome prediction after thrombectomy in patients with ischemic stroke
Source: PLoS One. 2020 Aug 13;15(8):e0236956. doi: 10.1371/journal.pone.0236956 (PMC7425881; doi:10.1371/journal.pone.0236956)
Supplement: S3 Table — (DOCX) [file pone.0236956.s003.docx]

| **S3 Table. Distribution of noncontrast CT ASPECTS (N=79)** | | |
| --- | --- | --- |
| **ASPECTS** | **Raw #** | **Frequency %** |
| 10 | 23 | 29.1% |
| 9 | 14 | 17.7% |
| 8 | 24 | 30.4% |
| 7 | 9 | 11.4 |
| 6 | 4 | 5.1% |
| 5 | 2 | 2.5% |
| 4 | 2 | 2.5% |
| 3 | 1 | 1.3% |
| 2 | 0 | 0% |
| 1 | 0 | 0% |
| 0 | 0 | 0% |
| Raw numbers and Frequency in percent of the Distribution of noncontrast CT ASPECTS. ASPECTS indicates Alberta Stroke Program early CT score; C, caudate nucleus; IC, internal capsule; INS, insula; L, lentiform nucleus; M1-M6, cortical regions of the ASPECTS | | |
