# Supplementary material for: Cerebral attenuation on single-phase CT angiography source images: Automated ischemia detection and morphologic outcome prediction after thrombectomy in patients with ischemic stroke
Source: PLoS One. 2020 Aug 13;15(8):e0236956. doi: 10.1371/journal.pone.0236956 (PMC7425881; doi:10.1371/journal.pone.0236956)
Supplement: S4 Table — (DOCX) [file pone.0236956.s004.docx]

| **S4 Table. Regional Distribution of Final Infarction in ASPECTS regions (N=79)** | | |
| --- | --- | --- |
| **ASPECTS region** | **Raw #** | **Frequency %** |
| C | 33 | 41.8% |
| IC | 22 | 27.8% |
| INS | 43 | 54.4% |
| L | 43 | 54.4% |
| M1 | 19 | 24.1% |
| M2 | 27 | 34.2% |
| M3 | 19 | 24.1% |
| M4 | 19 | 24.1% |
| M5 | 32 | 40.5% |
| M6 | 16 | 20.3% |
| Raw numbers and Frequency in percent of the Distribution of Final Infarction in ASPECTS regions. ASPECTS indicates Alberta Stroke Program early CT score; C, caudate nucleus; IC, internal capsule; INS, insula; L, lentiform nucleus; M1-M6, cortical regions of the ASPECTS | | |
