# Supplementary material for: Cerebral attenuation on single-phase CT angiography source images: Automated ischemia detection and morphologic outcome prediction after thrombectomy in patients with ischemic stroke
Source: PLoS One. 2020 Aug 13;15(8):e0236956. doi: 10.1371/journal.pone.0236956 (PMC7425881; doi:10.1371/journal.pone.0236956)
Supplement: S5 Table — (DOCX) [file pone.0236956.s005.docx]

| **S5 Table. Comparison of automated rHU measurements on CTASI and noncontrast CT for the Classification of regional ischemic core** | | | | | | | | | |
| --- | --- | --- | --- | --- | --- | --- | --- | --- | --- |
|  | | | | | | | | | |
| **Location** | **CTASI**  **AUC (95% CI)** | | **NCCT**  **AUC (95% CI)** | | **ROC comparison**  **P value** | | **CTASI**  **Accuracy** | **NCCT**  **Accuracy** | **Accuracy comparison**  **P value** |
|  | | | | | | | | | |
| **Classification of Regional Ischemic Core by CTASI rHU** | | | | | | | | | |
| C | 0.85 | (0.75-0.92) | 0.68 | (0.56-0.78) | | 0.05 | 78% | 86% | 0.02 |
| IC | 0.75 | (0.64-0.84) | 0.70 | (0.58-0.79) | | 0.48 | 75% | 67% | 0.03 |
| INS | 0.69 | (0.57-0.79) | 0.61 | (0.49-0.71) | | 0.34 | 67% | 61% | 0.06 |
| L | 0.87 | (0.77-0.93) | 0.77 | (0.66-0.86) | | 0.11 | 82% | 71% | 0.01 |
| M1 | 0.72 | (0.61-0.82) | 0.63 | (0.51-0.73) | | 0.29 | 78% | 77% | 1.00 |
| M2 | 0.70 | (0.59-0-80) | 0.55 | (0.44-0.66) | | 0.06 | 73% | 62% | 0.01 |
| M3 | 0.67 | (0.56-0.78) | 0.52 | (0.41-0.64) | | 0.18 | 72% | 59% | 0.01 |
| M4 | 0.68 | (0.57-0.78) | 0.65 | (0.53-0.75) | | 0.68 | 73% | 54% | <0.001 |
| M5 | 0.55 | (0.43-0.66) | 0.55 | (0.44-0.66) | | 0.90 | 53% | 57% | 0.25 |
| M6 | 0.75 | (0.63-0.84) | 0.61 | (0.50-0.72) | | 0.10 | 78% | 72% | 0.06 |
|  | | | | | | | | | |
| rHU was defined as the ratio of regional x-ray attenuation measurements of the ischemic to the non-ischemic hemisphere. Ischemic core was defined as ischemic change on the parametric cerebral blood flow map as well as cerebral blood volume map. Comparison of ROC curves was performed with the method of Hanley and McNeil. Comparison of test Accuracy was performed with McNemar´s Test. AUC indicates area under the curve values; C, caudate nucleus; CI, confidence interval; IC, internal capsule; INS, insula; L, lentiform nucleus; M1-M6, cortical regions of the ASPECTS score; rHU, relative Hounsfield Units; and ROC, receiver operating characteristics; ASPECTS, Alberta Stroke Program Early CT Score; CTASI, CT angiography source images; NCCT, non-contrast CT. P Values <0.05 indicate statistical significance. | | | | | | | | | |
