# Supplementary material for: Cerebral attenuation on single-phase CT angiography source images: Automated ischemia detection and morphologic outcome prediction after thrombectomy in patients with ischemic stroke
Source: PLoS One. 2020 Aug 13;15(8):e0236956. doi: 10.1371/journal.pone.0236956 (PMC7425881; doi:10.1371/journal.pone.0236956)
Supplement: S6 Table — (DOCX) [file pone.0236956.s006.docx]

| **S6 Table. ROC Analysis of CTASI-rHU Values for the Classification of Indicated Parameters** | | | | | | | | | |
| --- | --- | --- | --- | --- | --- | --- | --- | --- | --- |
|  |  |  |  |  |  |  |  |  |  |
| **Regional CTASI-rHU Values** | **AUC (95% CI)** | | | | **P Value** | **Youden's  Index** | **Associated  Cut-Off Value (rHU)** | **Associated Sensitivity** | **Associated Specificity** |
|  |  |  |  |  |  |  |  |  |  |
| **Classification of Final Infarction after Unsuccessful Reperfusion (mTICI 0-2a), n=17** | | | | | | | | | |
| C | 0.75 | (0.49-1.00) | | | 0.06 | 0.51 | 0.92 | 89% (8/9) | 63% (5/8) |
| IC | 0.67 | (0.33-1.00) | | | 0.33 | 0.43 | 0.88 | 60% (3/5) | 83% (10/12) |
| INS | 0.58 | (0.22-0.94) | | | 0.67 | 0.31 | 0.71 | 31% (4/13) | 100% (4/4) |
| L | 0.59 | (0.29-0.89) | | | 0.57 | 0.36 | 0.80 | 50% (5/10) | 86% (6/7) |
| M1 | 0.74 | (0.48-1.00) | | | 0.07 | 0.56 | 0.89 | 70% (7/10) | 86% (6/7) |
| M2 | 0.72 | (0.44-100) | | | 0.12 | 0.54 | 0.86 | 88% (7/8) | 67% (6/9) |
| M3 | 0.62 | (0.30-0.93) | | | 0.47 | 0.35 | 0.95 | 60% (4/5) | 75% (9/12) |
| M4 | 0.72 | (0.42-1.00) | | | 0.15 | 0.53 | 0.93 | 75% (6/8) | 78 % (7/9) |
| M5 | 0.72 | (0.47-0.97) | | | 0.09 | 0.50 | 0.84 | 50% (6/12) | 100% (5/5) |
| M6 | 0.82 | (0.58-1.00) | | | 0.01 | 0.63 | 0.94 | 80% (4/5) | 83% (10/12) |
|  | | | | | | | | | |
| **Classification of Final Infarction after Successful Reperfusion (mTICI 2b-3), n=62** | | | | | | | | | |
| C | 0.91 | (0.83-0.98) | | | <0.001 | 0.76 | 0.92 | 100% (24/24) | 76% (29/38) |
| IC | 0.72 | (0.58-0.85) | | | <0.001 | 0.40 | 0.95 | 88% (15/17) | 51% (23/45) |
| INS | 0.63 | (0.49-0.77) | | | 0.06 | 0.24 | 0.74 | 30% (9/30) | 94% (30/32) |
| L | 0.88 | (0.78-0.97) | | | <0.001 | 0.68 | 0.88 | 82% (27/33) | 86% (25/29) |
| M1 | 0.66 | (0.49-0.84) | | | 0.07 | 0.40 | 0.94 | 100% (9/9) | 39% (21/53) |
| M2 | 0.61 | (0.46-0.76) | | | 0.16 | 0.22 | 0.87 | 53% (10/19) | 70% (30 / 43) |
| M3 | 0.75 | (0.61-0.88) | | | <0.001 | 0.45 | 0.97 | 79% (12/14) | 67 % (32/48) |
| M4 | 0.62 | (0.43-0.82) | | | 0.21 | 0.24 | 0.95 | 46% (5/11) | 78% (40/51) |
| M5 | 0.52 | (0.36-0.68) | | | 0.79 | 0.19 | 0.96 | 95% (19/20) | 24% (10/42) |
| M6 | 0.67 | (0.51-0.84) | | | 0.04 | 0.31 | 0.99 | 100% (11/11) | 31% (16/51) |
|  | | | | | | | | | |
| rHU was defined as the ratio of regional x-ray attenuation measurements of the ischemic to the non-ischemic hemisphere. Ischemic core was defined as ischemic change on the parametric cerebral blood flow map as well as cerebral blood volume map. Sensitivity and specificity for the indicated cut-off value are presented as percentage and numbers as raw data in parentheses. AUC indicates area under the curve values; C, caudate nucleus; CI, confidence interval; IC, internal capsule; INS, insula; L, lentiform nucleus; M1-M6, cortical regions of the ASPECTS score; rHU, relative Hounsfield Units; and ROC, receiver operating characteristics; mTICI, modified Treatment in Cerebral Ischemia Score. P Values <0.05 indicate statistical significance. | | | | | | | | | |
