# Supplementary material for: Cerebral attenuation on single-phase CT angiography source images: Automated ischemia detection and morphologic outcome prediction after thrombectomy in patients with ischemic stroke
Source: PLoS One. 2020 Aug 13;15(8):e0236956. doi: 10.1371/journal.pone.0236956 (PMC7425881; doi:10.1371/journal.pone.0236956)
Supplement: S7 Table — (DOCX) [file pone.0236956.s007.docx]

| **S7 Table. Distribution of Collateral Scores** | | | | | | |
| --- | --- | --- | --- | --- | --- | --- |
| Score | **0** | **1** | **2** | **3** | **4** | **5** |
| Collateral Status by Maas et al. (3) | NA | 8 (10.1%) | 61 (77.2%) | 10 (12.7%) | 0 (0%) | 0 (0%) |
| Collateral Status by  Tan et al. (4) | 8 (10.1%) | 30 (38.0%) | 31 (39.2%) | 10 (12.7%) | NA | NA |
| Distribution of collateral scores in the study cohort displayed as raw number (percentage). Scale by Maas et al.:1=absent, 2=less than contralateral side, 3=equal to contralateral side, 4=more than contralateral side, 5=exuberant. Scale by Tan et al.: 0=absent, 1=collateral filling ≤50% but >0%, 2= collateral filling ≥ 50% but < 100%, 3= 100% collateral filling. NA indicates not applicable. | | | | | | |
