# Supplementary material for: Cerebral attenuation on single-phase CT angiography source images: Automated ischemia detection and morphologic outcome prediction after thrombectomy in patients with ischemic stroke
Source: PLoS One. 2020 Aug 13;15(8):e0236956. doi: 10.1371/journal.pone.0236956 (PMC7425881; doi:10.1371/journal.pone.0236956)
Supplement: S8 Table — (DOCX) [file pone.0236956.s008.docx]

| **S8 Table. Linear Regression Analysis for the Association of Hemispheric CTASI-rHU and Collateral Status by Maas et al. (3)** | | |
| --- | --- | --- |
| N=79 | **β** | **p value** |
| Collateral Status by Maas et al. | 0.052 | 0.61 |
| Total Ischemic Volume | -0.648 | <0.001 |
| Ischemic Core Volume | 0.148 | 0.22 |
| Noncontrast CT ASPECTS | 0.183 | 0.085 |
| Multivariate linear regression analyses were performed for the indicated imaging parameters as independent variables and hemispheric CTASI-rHU as dependent variable. CTASI indicates CT angiography source images; rHU, relative Hounsfield Units; ASPECTS, Alberta Stroke Program Early CT Score. p<0.05 indicates statistical significance. | | |
