# Supplementary material for: Cerebral attenuation on single-phase CT angiography source images: Automated ischemia detection and morphologic outcome prediction after thrombectomy in patients with ischemic stroke
Source: PLoS One. 2020 Aug 13;15(8):e0236956. doi: 10.1371/journal.pone.0236956 (PMC7425881; doi:10.1371/journal.pone.0236956)
Supplement: S10 Table — (DOCX) [file pone.0236956.s010.docx]

| **S10 Table. Agreement of Automated and Visual CTASI ASEPCTS** | | | |
| --- | --- | --- | --- |
|  | **Intraclass Correlation Coefficient** | **(95%-CI)** | **Interpretation** |
| CBF ASPECTS vs. Visual CTASI ASPECTS | 0.58 | (0.35-0.73) | moderate |
| CBV ASPECTS vs. Visual CTASI ASPECTS | 0.57 | (0.33-0.73) | moderate |
| CBF ASPECTS vs. Ischemia Weighted Automated CTASI ASPECTS | 0.77 | (0.64 – 0.85) | good |
| CBF ASPECTS vs. Core Weighted Automated CTASI ASPECTS | 0.60 | (0.37 – 0.74) | moderate |
| CBV ASPECTS vs. Ischemia Weighted Automated CTASI ASPECTS | 0.56 | (0.31-0.72) | moderate |
| CBV ASPECTS vs. Core Weighted Automated CTASI ASPECTS | 0.57 | (0.33-0.73) | moderate |
| Ischemia Weighted Automated vs. Visual CTASI ASPECTS | 0.79 | (0.67-0.89) | good |
| Core Weighted Automated vs. Visual CTASI ASPECTS | 0.79 | (0.66-0.86) | good |
| Measurements of agreement for the indicated parameters. Extent of hypoattenuation on CTASI as well as on CBF and CBV maps was determined by consensus reading. ICC Interpretation: <0.5 – poor, 0.5-0.75 – moderate, >0.75 – good (2). ASPECTS indicates Alberta Stroke Program Early CT Score; CBF, cerebral blood flow; CBV, cerebral blood volume; CTASI; CT angiography source images; CI, confidence interval; ICC, intraclass correlation coefficient. | | | |
