# Supplementary material for: Cerebral attenuation on single-phase CT angiography source images: Automated ischemia detection and morphologic outcome prediction after thrombectomy in patients with ischemic stroke
Source: PLoS One. 2020 Aug 13;15(8):e0236956. doi: 10.1371/journal.pone.0236956 (PMC7425881; doi:10.1371/journal.pone.0236956)
Supplement: S1 File — (DOCX) [file pone.0236956.s011.docx]

**Supporting Material References**

1. Fiebach, J. B., et al. (2002). "CT and diffusion-weighted MR imaging in randomized order: diffusion-weighted imaging results in higher accuracy and lower interrater variability in the diagnosis of hyperacute ischemic stroke." Stroke 33(9): 2206-2210.

2. Koo, T. K. and M. Y. Li (2016). "A Guideline of Selecting and Reporting Intraclass Correlation Coefficients for Reliability Research." J Chiropr Med 15(2): 155-163.”

3. Maas MB, Lev MH, Ay H, Singhal AB, Greer DM, Smith WS, et al. Collateral vessels on CT angiography predict outcome in acute ischemic stroke. Stroke. 2009;40(9):3001-5.

4. Tan JC, Dillon WP, Liu S, Adler F, Smith WS, Wintermark M. Systematic comparison of perfusion-CT and CT-angiography in acute stroke patients. Ann Neurol. 2007;61(6):533-43.
